# Attenuation of Phosphorylation-dependent Activation of Cystic Fibrosis Transmembrane Conductance Regulator (CFTR) by Disease-causing Mutations at the Transmission Interface*

**DOI:** 10.1074/jbc.M116.762633

**Published:** 2016-12-21

**Authors:** Stephanie Chin, Donghe Yang, Andrew J. Miles, Paul D. W. Eckford, Steven Molinski, B. A. Wallace, Christine E. Bear

**Affiliations:** From the ‡ Programme of Molecular Structure and Function, Hospital for Sick Children, Toronto M5G 0A4, Canada,; the § Department of Biochemistry, University of Toronto, Toronto, Canada,; the ¶ Institute of Structural and Molecular Biology, Birkbeck College, University of London, London WC1E 7HX, United Kingdom, and; the ‖ Department of Physiology, University of Toronto, Toronto, Canada

**Keywords:** cysteine-mediated cross-linking, cystic fibrosis transmembrane conductance regulator (CFTR), ion channel, phosphorylation, spectroscopy, synchrotron radiation circular dichroism

## Abstract

Cystic fibrosis transmembrane conductance regulator (CFTR) is a multidomain membrane protein that functions as a phosphorylation-regulated anion channel. The interface between its two cytosolic nucleotide binding domains and coupling helices conferred by intracellular loops extending from the channel pore domains has been referred to as a transmission interface and is thought to be critical for the regulated channel activity of CFTR. Phosphorylation of the regulatory domain of CFTR by protein kinase A (PKA) is required for its channel activity. However, it was unclear if phosphorylation modifies the transmission interface. Here, we studied purified full-length CFTR protein using spectroscopic techniques to determine the consequences of PKA-mediated phosphorylation. Synchrotron radiation circular dichroism spectroscopy confirmed that purified full-length wild-type CFTR is folded and structurally responsive to phosphorylation. Intrinsic tryptophan fluorescence studies of CFTR showed that phosphorylation reduced iodide-mediated quenching, consistent with an effect of phosphorylation in burying tryptophans at the transmission interface. Importantly, the rate of phosphorylation-dependent channel activation was compromised by the introduction of disease-causing mutations in either of the two coupling helices predicted to interact with nucleotide binding domain 1 at the interface. Together, these results suggest that phosphorylation modifies the interface between the catalytic and pore domains of CFTR and that this modification facilitates CFTR channel activation.

## Introduction

Cystic fibrosis is caused by mutations of an ATP binding cassette (ABC) anion channel, cystic fibrosis transmembrane conductance regulator (CFTR).^2^ CFTR has two nucleotide binding domains (NBDs), two membrane-spanning domains (MSDs), and a regulatory (R) domain (1). The interface between the NBDs and MSDs of CFTR is an α-helical region consisting of intracellular loops (ICLs) and coupling helices (CHs) (1). The interface has been referred to as the transmission interface between the pore and catalytic domains of CFTR (2).

Our understanding of the molecular basis for phosphorylation-dependent regulation of channel activation has evolved considerably, and we have gained greater insight into its complexity. The phosphorylation-regulated R domain of CFTR is a flexible, disordered region that undergoes dynamic interactions with other CFTR domains (3, 4). Phosphorylation of the R domain is thought to alter multiple interactions with other CFTR domains, with certain interactions becoming weaker (4) and other interactions acquiring higher affinities (5, 6). The regulation of domain-domain interactions has been studied using isolated domains and in chemical cross-linking studies of a full-length cys-less CFTR protein that has all of its native cysteine residues mutated to other residues with strategically inserted cysteine pairs. For example, studies of isolated CFTR domains suggest that phosphorylation of the phospho-regulated insertion within NBD1 (the regulatory insertion (RI)) decreases its interaction with the core of NBD1, and this in turn acts allosterically to promote interaction between CH1 of ICL1 and NBD1 at the transmission interface (4, 7).

Cysteine cross-linking studies in the full-length protein showed that phosphorylation enhanced NBD1-NBD2 dimerization (1, 8). Studies of the consequences of strategic single site mutations at the dimer interface supported the importance of this phosphorylation-regulated interface in channel activation (9, 10). In contrast to the above predictions based on isolated domains, chemical cross-linking studies of the full-length CFTR failed to show modification of the interaction between the CHs and the NBDs by phosphorylation (1, 8). These findings do not support the previous hypothesis that this transmission interface between the pore and catalytic domains is regulated by phosphorylation. On the other hand, the previous hypothesis was supported by a recent study that showed that an isolated peptide derived from ICL1 disrupted phosphorylation-dependent activation of full-length CFTR (11). Hence, there is considerable uncertainty regarding the regulation of the transmission interface by phosphorylation, highlighting the need to develop new approaches for its study in the context of the full-length protein.

The goal of this study was to employ biophysical approaches that would permit insight into dynamic conformational changes caused by protein kinase A (PKA) phosphorylation in the purified full-length wild-type (WT) CFTR protein. Furthermore, the relative importance of the CHs in phosphorylation-dependent channel activation was probed in comparative studies of disease-causing mutations in these regions.

## Results

### 

#### 

##### Purified Full-length WT-CFTR Is Properly Folded, and Its Structure Is Modified by PKA Phosphorylation

**FIGURE 1.**
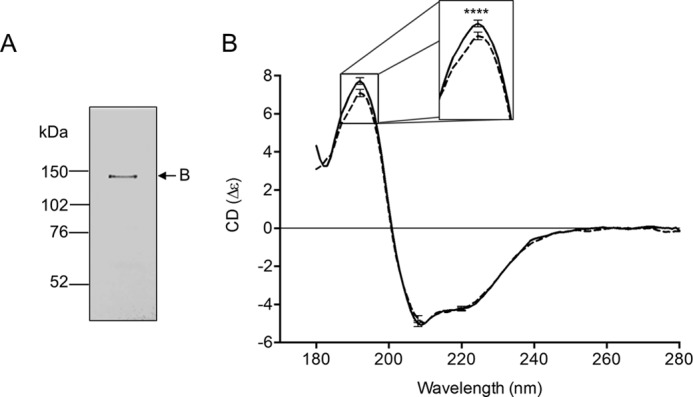
**PKA phosphorylation modifies the secondary structure of purified full-length WT-CFTR.** *A*, silver stained gel shows the purity of the full-length WT-CFTR and that it runs at its expected molecular weight of around 140–150 kDa in its core-glycosylated state (band *B*), the only form of CFTR that is expressed in the *Sf9* expression system. *B*, the scaled delta epsilon (Δϵ) SRCD spectra show that purified full-length WT-CFTR is mostly α-helical with characteristic negative peaks at 209 and 222 nm and a positive peak at 192 nm. SRCD spectra for untreated purified full-length WT-CFTR in DDM micelles (*hashed line*) and PKA-phosphorylated CFTR (*solid line*) are shown. *Error bars* indicate ± 1 S.D. between a total of six spectra from three replicate scans of two aliquots of sample from the same purification. The *inset* shows that phosphorylation significantly increases the peak at 192 nm (****, *p* < 0.0001, *t* test) relative to that at 222 nm.

Full-length WT-CFTR was purified after expression in *Sf9* membranes as previously described (12). Briefly, CFTR was extracted using the detergent, fos-choline-14, and CFTR (bearing a polyhistidine tag) was partially purified by virtue of the affinity of this tag to the Ni-NTA resin (12). Fos-choline-14 was replaced with *n*-dodecyl β-d-maltoside (DDM), and the protein-detergent complex was eluted from the affinity matrix (12). The purified protein was treated with 200 nm PKA and 5 mm Mg-ATP on the Ni-NTA column to enable extensive washing as described (12), and phosphorylation at multiple consensus sites was confirmed previously using selected reaction monitoring mass spectrometry (13). Our methods were successful in purifying full-length WT-CFTR as shown by the single, silver-stained protein band running at the expected molecular mass of ∼140–150 kDa for the core-glycosylated form (band *B*) of CFTR expressed in *Sf9* cells (Fig. 1*A*). In a subset of our experiments, we confirmed that the purified full-length WT-CFTR is functional as a phosphorylation and ATP-regulated anion channel upon reconstitution in proteoliposomes using our previously published iodide efflux protocols (12, 14).

Synchrotron radiation circular dichroism (SRCD) spectroscopy was used to examine WT-CFTR. The spectra resembled that of a protein with a high helical content, as expected with negative peaks at 209 and 222 nm and a positive peak at 192 nm (Fig. 1*B*). Furthermore, comparison of the spectra of non-PKA-phosphorylated WT-CFTR and PKA-phosphorylated WT-CFTR indicated a subtle yet significant increase in the peak at 192 nm that corresponds to an increase in helical structure content (Fig. 1*B*). Indeed, the ratio of the magnitudes of the 192- and 222-nm peaks increased from 1.74 in the non-PKA-phosphorylated state to 1.86 after PKA phosphorylation, a clear indication that the shapes of the curves are different and that the observed differences do not simply arise from different magnitudes of the spectra (15). Previous nuclear magnetic resonance (NMR) and CD spectroscopy studies on the isolated NBD1 and the R region (residues 654–838) have found that phosphorylation decreased the helical content of those isolated domains (4, 16). Thus, the increase in helical structure upon PKA phosphorylation observed by SRCD in the full-length WT-CFTR is likely due to allosteric effects induced by phosphorylation of the RI and R region (1, 17).

**FIGURE 2.**
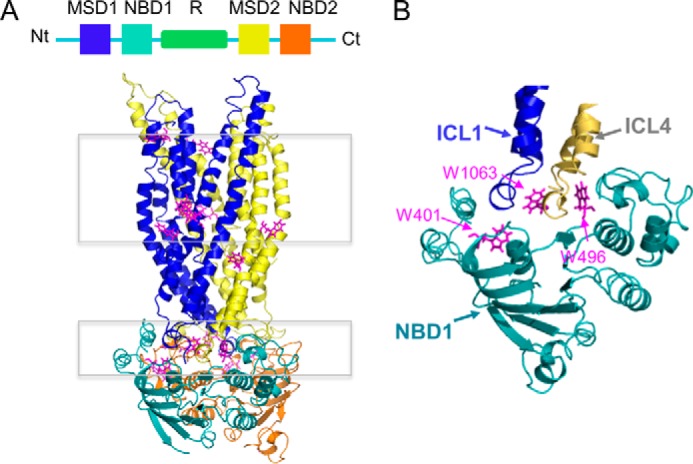
**PKA phosphorylation is proposed to modify the transmission interface, as determined by intrinsic tryptophan fluorescence studies.** *A*, *top*, linear arrangement of CFTR domains from the N-terminal (*Nt*) to C-terminal (*Ct*) end, which shows the membrane spanning domain 1 (*MSD1*) in *blue*, nucleotide binding domain 1 (*NBD1*) in *teal*, regulatory (*R*) domain in *green*, MSD2 in *yellow*, and NBD2 in *orange. Bottom*, the tryptophan residues on a CFTR homology model based on a bacterial transporter, Sav1866 (17), are shown as *pink sticks* and are mostly located at the membrane-solvent interface (with the membrane indicated by a *rectangle*) and intracellular loop (*ICL*)-NBD interface, *i.e.* the transmission interface (indicated by a *distinct rectangle below the membrane*). The R domain is omitted from this homology model of CFTR. *B*, close-up view of the transmission interface with NBD1 of the CFTR homology model (17) as this interface has been studied extensively with regard to its role in channel activation. This shows that the transmission interface contains the CHs of the ICLs and NBD1 with multiple tryptophans (*i.e.* 401, 496, and 1063) shown as *pink sticks* that are located at the interface between NBD1 (*teal*) and ICL1 (*blue*) or ICL4 (*yellow*).

**FIGURE 3.**
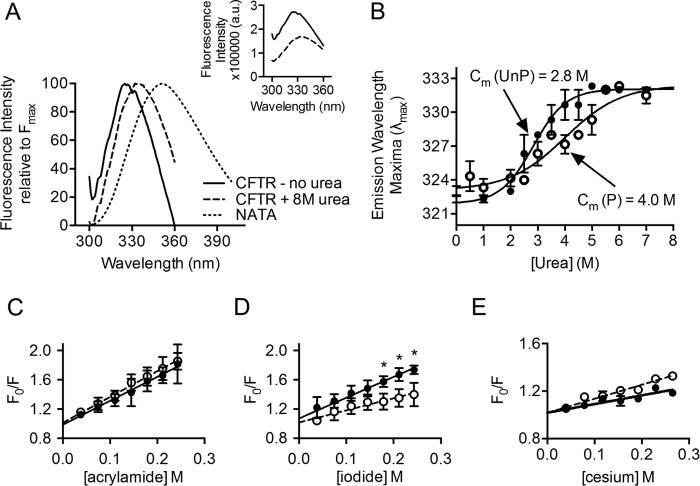
**PKA phosphorylation modifies the tertiary structure of purified full-length WT-CFTR, possibly within an electrostatic region.** *A*, representative intrinsic tryptophan fluorescence readouts of PKA-phosphorylated full-length WT-CFTR before and after urea denaturation that were normalized to fluorescence maxima (*F*_max_). The intrinsic tryptophan fluorescence of PKA-phosphorylated full-length WT-CFTR (*solid line*) exhibits a red shift in λ_max_ upon treatment with the final concentration of 8 m urea (*dashed line*). CFTR was not completely denatured by 8 m urea, as the trace did not completely overlap with the NATA trace (*dotted line*) in which all tryptophans are exposed to the solvent. *Inset*: raw fluorescence intensity of untreated purified full-length WT-CFTR protein (*solid line*) and treated protein with 8 m urea (*dashed line*) shows that urea shifts the λ_max_ and decreases the fluorescence intensity of purified CFTR. *B*, comparing the red shift in λ_max_ across urea concentrations of PKA-phosphorylated (*open circles*) and non-PKA-phosphorylated (*closed circles*) full-length CFTR shows that the PKA-phosphorylated WT-CFTR is less susceptible to urea denaturation compared with non-PKA-phosphorylated CFTR. These two denaturation curves are significantly different; non-PKA-phosphorylated WT-CFTR has a *C_m_* of 2.8 m urea, whereas PKA-phosphorylated WT-CFTR has a *C_m_* of 4.0 m urea. The *m* value or slope of unfolding was also lower in the PKA-phosphorylated sample in which the *m* value was 0.8 kcal/mol·m^−1^ in the non-PKA-phosphorylated WT-CFTR compared with 0.6 kcal/mol·m^−1^ in the PKA-phosphorylated WT-CFTR. *Error bars* indicate ± 1 S.D. between replicate samples (*n* = 5 biological replicates, and *n* = 5 technical replicates, *p* = 0.0057, two-way analysis of variance). *C–E*, Stern-Volmer plots of tryptophan quenching of purified full-length non-PKA-phosphorylated (*closed circles*) and PKA-phosphorylated (*open circles*) WT-CFTR at the major intrinsic tryptophan fluorescence peak, obtained from excitation at 290 nm and emission at 322 nm, were generated. *C*, quenching with acrylamide of purified full-length non-PKA-phosphorylated (*closed circles*) and PKA-phosphorylated (*open circles*) WT-CFTR shows no difference upon PKA phosphorylation (*n* = 3 biological replicates and *n* = 3 technical replicates, *p* > 0.05, multiple *t* tests using the Holm-Sidak method). *D*, quenching of purified full-length non-PKA-phosphorylated (*closed circles*) and PKA-phosphorylated (*open circles*) WT-CFTR with negatively charged iodide was significantly reduced upon PKA phosphorylation at high iodide concentrations starting at 0.18 m iodide (*n* = 3 biological replicates and *n* = 3 technical replicates; *, *p* < 0.005, Multiple *t* tests using the Holm-Sidak method). *E*, interestingly, quenching of purified full-length non-PKA-phosphorylated (*closed circles*) and PKA-phosphorylated (*open circles*) WT-CFTR with positively charged cesium (*n* = 3 biological replicates and *n* = 3 technical replicates) showed an opposite quenching effect from iodide upon PKA phosphorylation, suggesting that phosphorylation modifies an electrostatic environment of the protein. *Error bars* indicate ± 1 S.D. between replicate samples.

Previous intrinsic tryptophan fluorescence studies of the *Escherichia coli* vitamin B_12_ ABC transporter, BtuCD, have shown that the interface between the CHs conferred by the MSD subunits (BtuC) and the NBD subunits (BtuD) was sensitive to urea-mediated unfolding (18, 19). According to the CFTR homology model based on the structure of Sav1866 that was generated by Dalton *et al.* (17), the tryptophan residues endogenous to CFTR reside at the membrane-solvent interface and at the transmission (ICL-NBD) interface (Fig. 2, *A* and *B*). Thus, we were prompted to determine whether measurements of intrinsic tryptophan fluorescence of purified full-length WT-CFTR could reveal phosphorylation-associated changes in urea-mediated unfolding. DDM-solubilized full-length WT-CFTR exhibited a decrease in fluorescence intensity (Fig. 3*A*, *inset*) and a red shift in emission wavelength maxima (λ_max_) with increasing concentrations of urea, from its initial wavelength of 322 nm to its final wavelength of 332 nm (Fig. 3, *A* and *B*). The decrease in fluorescence intensity and red shift in the λ_max_ reported the change in the chemical environment of the tryptophans upon urea-induced unfolding of the protein. Interestingly, this red shift did not reach the λ_max_ of 355 nm observed in an *N*-acetyl-l-tryptophanamide (NATA) sample of free soluble tryptophan analogues (Fig. 3*A*), suggesting that urea failed to completely unfold CFTR. Hence, akin to the previous SRCD experiments, intrinsic tryptophan fluorescence studies also demonstrated that the purified protein is properly folded in DDM micelles.

PKA phosphorylation shifted the concentration dependence for urea-induced unfolding to the right, from a midpoint urea concentration (*C_m_*) of ∼2.8 m in the non-PKA-phosphorylated sample to a *C_m_* of ∼4.0 m in the PKA-phosphorylated sample (Fig. 3*B*). In addition, the slope of the transition known as the *m* value was lower in the PKA-phosphorylated condition: the *m* value was 0.8 kcal/mol·m^−1^ in non-PKA-phosphorylated WT-CFTR compared with 0.6 kcal/mol·m^−1^ in PKA-phosphorylated WT-CFTR (Fig. 3*B*). These findings suggest that PKA phosphorylation protected the chemical environment of endogenous tryptophans from urea-induced unfolding at either the membrane-solvent interface or at the ICL-NBD interface (Fig. 2, *A* and *B*).

To determine whether the intrinsic tryptophan fluorescence measurements reported the effect of PKA phosphorylation on the membrane-solvent interface or on the ICL-NBD interface, fluorescence quenching studies were conducted to probe the environment of the tryptophans that were responsible for the intrinsic tryptophan fluorescence of purified full-length WT-CFTR. Quenchers of different polarity and charge were tested that include polar and uncharged acrylamide, negatively charged iodide, and positively charged cesium salt. As expected, NATA was readily quenched by all quenchers with high Stern-Volmer constant (*K*_sv_) values (Table 1), as the tryptophan analogues were completely exposed to the solvent and were, therefore, highly accessible to the quenchers. In contrast, tryptophans intrinsic to full-length WT-CFTR were relatively resistant to quenching with significantly lower *K*_sv_ values compared with NATA, as expected for a properly folded protein (Table 1). Interestingly, all quenchers reduced the intrinsic tryptophan fluorescence of purified full-length WT-CFTR in a linear fashion, suggesting that only one cluster of tryptophans was reported for each quencher of this assay (Fig. 3, *C–E*).

**TABLE 1:** **Stern-Volmer constants of tryptophan fluorescence quenching studies of NATA, non-PKA, and PKA phosphorylated Wt-CFTR** Summary of the Stern-Volmer constants (*K*_sv_) values of NATA, non-PKA-phosphorylated, and PKA-phosphorylated Wt-CFTR with the quenchers: acrylamide, iodide, and cesium. The *K*_sv_ values of all quenchers were significantly higher in the NATA sample compared with purified full-length Wt-CFTR. The *K*_sv_ values of non-PKA-phosphorylated and PKA-phosphorylated Wt-CFTR with acrylamide were not different outside the range of error, whereas the *K*_sv_ values with iodide and cesium were different outside the range of error. Based on these results, we propose that intrinsic tryptophan fluorescence may be reporting the tryptophans at the ICL-NBD interface.

Sample	*K*_sv_		
	Acrylamide	Iodide	Cesium
	m^−*1*^		
NATA	23.57 ± 2.66	15.81 ± 0.54	3.58 ± 0.10
Non-PKA-phosphorylated CFTR	3.49 ± 0.41	2.85 ± 0.22	0.73 ± 0.22
PKA-phosphorylated CFTR	3.53 ± 0.22	1.89 ± 0.34	1.18 ± 0.38

Quenching with polar and uncharged acrylamide was not affected by the phosphorylation status of CFTR (Fig. 3*C*). We propose that acrylamide quenching may be reporting the membrane-solvent interface due to its difficulty to diffuse across the membrane (20). In contrast, quenching with charged quenchers, iodide and cesium, were dependent on its phosphorylation status (Fig. 3, *D* and *E*). PKA phosphorylation of purified CFTR led to a lower *K*_sv_ value upon quenching with negatively charged iodide, whereas it led to a higher *K*_sv_ value upon quenching with positively charged cesium (Table 1). Based on their properties, the charged quenchers may be reporting the cluster of tryptophans at the cytosol. In addition, the negative charge conferred by phosphorylation may have modulated the propensity of the reporting tryptophans to be quenched by aqueous, charged agents. Interestingly, there is a phosphorylation site, Ser(P)-422, on the RI (that Lewis *et al.* (21) report as residues 413–428) but Aleksandrov *et al.* (22) report as residues 404–435) of NBD1 that is proximal to the ICL-NBD1 interface (4, 7, 23, 24). Previous NMR studies have suggested that phosphorylation at Ser(P)-422 enhanced interactions at the ICL1-NBD1 and ICL4-NBD1 interfaces (4, 7, 23). Based on these results, we propose that PKA phosphorylation may be modifying the electrostatic environment of the ICL-NBD1 interface as there are multiple tryptophans (including residues 401, 496, and 1063) residing in proximity to the putative transmission interface consisting of CH1, CH4, and NBD1 that may be reported in the intrinsic tryptophan fluorescence studies (Fig. 2*B*).

##### Cysteine Cross-linking Studies Using a Membrane-permeable Sulfydryl Modifying Reagent Confirmed a Physical Interaction between CH4 of ICL4 and NBD1

**FIGURE 4.**
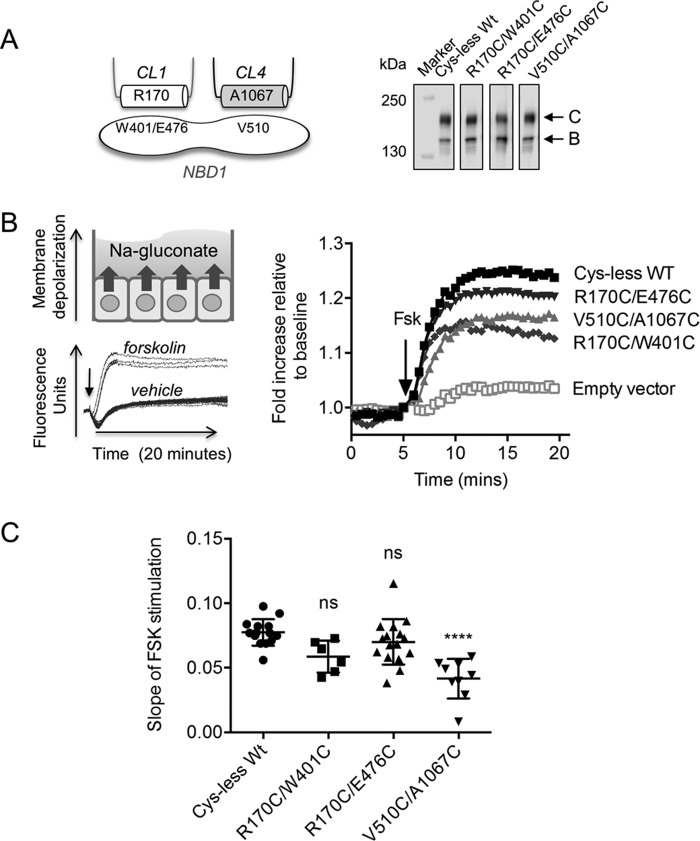
**Cys-less CFTR mutants are functionally competent and introduction of V510C/A1067C at the ICL4-NBD1 interface affects phosphorylation-dependent channel activity.** *A*, *left*, a schematic showing the location of the residues predicted to interact at the ICL-NBD1 interfaces: Val-510 on NBD1 and Ala-1067 on CH4 at the ICL4-NBD1 interface and Arg-170 on CH1 and Trp-401/Glu-476 on NBD1 at the ICL1-NBD1 interface. Cysteine pairs were generated at those residues to investigate interactions at those interfaces with cysteine cross-linking studies. *Right*, the immunoblot shows that the cys-less WT-CFTR mutants were expressed at the endoplasmic reticulum as core-glycosylated protein (represented by band *B*) and at the cell surface as complex-glycosylated protein (represented by band *C*). *B*, *Left*, schematic of the basis of the FLIPR response of transiently transfected HEK-293 GripTite cells in sodium (*Na-*) gluconate buffer. Activation of CFTR channel by forskolin leads to membrane depolarization of the cells, which corresponds to a significant increase in fluorescence intensity compared with vehicle. *Right*, CFTR mutants were stimulated with the cAMP agonist, Fsk (10 μm), after 5 min of baseline reading. Cys-less WT along with V510C/A1067C, R170C/W401C, and R170C/E476C cys-less WT-CFTR channels were found to retain partial channel activity after forskolin stimulation in the FLIPR assay. The empty vector was transfected in HEK-293 GripTite cells as a negative control. *C*, the initial slope within the first 3 min of forskolin stimulation was significantly lower in V510C/A1067C, whereas R170C/W401C and R170C/E476C did not have a significantly different rate compared with cys-less WT. *Error bars* indicate ±1 S.D. between replicate samples (*n* = 9 biological replicates and *n* = 3 technical replicates, *p* < 0.0001 between cys-less WT and V510C/A1067C, one-way analysis of variance). *ns*, not significant.

Previous studies investigating phosphorylation-dependent changes in the affinity of the CHs and NBD1 used chemical cross-linking of cysteine pairs introduced on opposing surfaces of NBD1 and CH1 or CH4 (Fig. 4*A*); cysteine pairs were introduced at positions previously shown to reside at these domain-domain interfaces in a CFTR construct lacking native cysteines (cys-less CFTR) (1, 8). These cross-linkers were long and membrane-impermeable, necessitating the application of these sulfydryl reagents to inside-out membrane vesicles (1, 8). In the current work we were prompted to determine if phosphorylation-dependent changes in cysteine cross-linking could be detected using the short (8 Å) and membrane-permeable maleimide reagent, bismaleimidoethane (BMOE), as this was previously successful in capturing phosphorylation-dependent changes of the NBD1-NBD2 interface in full-length cys-less CFTR (10).

First, we confirmed that the cysteine mutants were expressed at the cell surface in human embryonic kidney (HEK)-293 GripTite cells as shown by the presence of complex-glycosylated form of the protein (band *C*) in the immunoblot (Fig. 4*A*). In addition, CFTR activation of the cysteine mutants was studied using the membrane potential plate reader assay: FLIPR (25). As shown in Fig. 4*B*, all of the cysteine mutants were functionally expressed as cAMP-activated conductances. Interestingly, the overall rate of activation was significantly reduced in V510C/A1067C cys-less CFTR (Fig. 4*C*). This defect could reflect a disulfide bond formation for the pair of cysteines introduced at CH4 and NBD1, which could account for attenuation of channel activity by modifying important dynamic conformational changes as previously suggested (1, 8).

**FIGURE 5.**
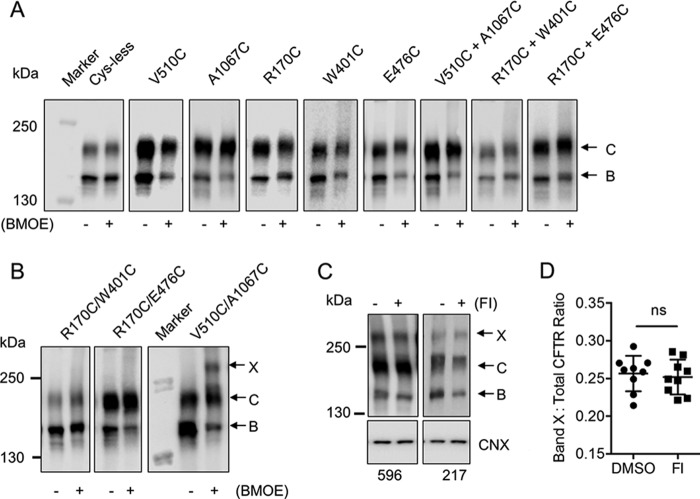
**Cysteine cross-linking was observed at the ICL4-NBD1 but not the ICL1-NBD1 interface, and this interaction was phosphorylation-independent.** *A*, immunoblot of negative controls consisting of cys-less WT-CFTR (*Cys-less*), single cysteine mutants (*V510C*, *A1067C*, *R170C*, *W401C*, and *E476C*), and co-transfected single cysteine mutants on different plasmids (*V510C*+*A1067C*, *R170C*+*W401C*, *R170C*+*E476C*) were treated with (+) and without (−) BMOE for 1 h at 27 °C. No additional bands except for bands representing the core-glycosylated protein (band *B*) and complex-glycosylated protein (band *C*) were found from the negative controls. *B*, immunoblot shows that a higher molecular mass band above 250 kDa, which corresponds to the cross-linked product of band *C* of the protein (band *X*), was only present upon treatment with BMOE on the construct expressing V510C and A1067C within the same CFTR protein (V510C/A1067C). This band was not present in the ICL1-NBD1 cysteine pairs, R170C/W401C and R170C/E476C. This suggests the BMOE was able to capture the interaction of the cysteine pairs at the ICL4-NBD1 interface but not at the ICL1-NBD1 interface. *C*, immunoblot shows that cross-linked V510C/A1067C was efficiently phosphorylated as shown by a decrease in signal after Fsk/3-isobutyl-1-methylxanthine (IBMX (*FI*) treatment and detection by a phosphorylation-sensitive antibody, 217 (anti-Ser-813 of human CFTR), compared with the non-phosphorylation sensitive CFTR antibody, 596 (epitope on NBD2 of human CFTR) (*n* = 3 biological replicates and *n* = 3 technical replicates). *D*, densitometry analysis of band *X* to total CFTR ratio showed no significant difference between the cysteine cross-linking of DMSO and Fsk/3-isobutyl-1-methylxanthine pretreatment of V510C/A1067C. *Error bars* indicate ± 1 S.D. between replicate samples (*n* = 9 biological replicates and *n* = 3 technical replicates, *p* = 0.7538, paired *t* test). *ns*, not significant.

As in previous studies by Mense *et al.* (10), we studied cross-linking of cysteine pairs in cys-less CFTR using a cell-permeable maleimide cross-linker, BMOE. Previous studies have shown that V510C on NBD1 and A1067C on CH4 were in close proximity and could be cross-linked with a methane-thiosulfonate reagent in membrane vesicles (8, 26). Thus, we studied those substitutions to confirm the efficacy of BMOE in capturing such interactions in intact cells. A unique molecular weight band (band *X*) above 250 kDa only appeared with BMOE treatment of cells expressing cys-less CFTR bearing two cysteines in the same protein (V510C/A1067C) (Fig. 5*B*) and not in cells expressing cys-less CFTR with single cysteines (V510C or A1067C) or co-expressing single cysteines from different cys-less CFTR constructs (*V510C*+*A1067C*) (Fig. 5*A*). This finding confirmed that the membrane-permeable cross-linker, BMOE, can effectively cross-link interacting residues within CFTR.

Previous studies have suggested that Arg-170 of CH1 interacts with residues of NBD1 (*i.e.* 402, 403, or 407) (1, 27). From our intrinsic tryptophan fluorescence studies, Trp-401 was a residue of interest that may be in close proximity to the transmission interface (Fig. 2*B*). Thus, the R170C/W401C mutant was generated to study the predicted interaction of those residues at the ICL1-NBD1 interface. Band *X* was not detected upon BMOE addition to cells expressing R170C/W401C and single cysteine mutants (R170C or W401C) or co-expressing single cysteines from different cys-less CFTR constructs (R170C+W401C) (Fig. 5, *A* and *B*). We have also applied these studies on a different site of NBD1 (by engineering a cysteine at Glu-476), which was also predicted to interact with Arg-170 of CH1 (27), to further interrogate the ICL1-NBD1 interface. The addition of BMOE to cells expressing R170C/E476C also failed to confer the appearance of the unique cross-linked band in SDS analyses (Fig. 5*B*). We did detect a modest increase in the apparent mass of band *B* of R170C/W401C and R170C/E476C cys-less CFTR after treatment with BMOE (Fig. 5*B*). However, we also observed a similar shift in CFTR protein bearing only one cysteine, such as R170C (Fig. 5*A*). We suggest that this change in mass does not represent cross-linking but, rather, a change in conformation induced by BMOE modification of a single cysteine. Hence, the membrane-permeable sulfydryl reagent, BMOE, was successful in capturing CH4-NBD1 but not CH1-NBD1 interactions in this study.

We then applied cysteine cross-linking studies to test our hypothesis that PKA phosphorylation modifies the ICL-NBD1 interfaces. We could only test the effect of phosphorylation on the ICL4-NBD1 interface as we could not detect cysteine cross-linking at the ICL1-NBD1 interface as previously shown (Fig. 5*B*). Phosphorylation has been shown to significantly enhance both band *B* and band *C* of WT-CFTR over time (28). To address this effect, we acutely treated the V510C/A1067C protein with cAMP agonists, forskolin (Fsk) and 3-isobutyl-1-methylxanthine, for 30 min and conducted cysteine cross-linking on ice. We did not detect a change in the relative abundance of band *X* between V510C and A1067C after PKA-mediated phosphorylation (Fig. 5, *C* and *D*). In separate experiments we also showed that band *X* did not appear in studies of R170C/(W410C or E476C) after phosphorylation with the cAMP agonists (data not shown). Together, these chemical cross-linking results were consistent with previous studies that suggest that chemical cross-linking may not be a sufficiently sensitive approach to detect subtle interactions mediated by PKA phosphorylation at the ICL-NBD1 interfaces (1, 8). Alternatively, it may be necessary to introduce cysteines at different positions in the cys-less CFTR protein to monitor phosphorylation-dependent changes in domain-domain affinity. For example, Corradi *et al.* (2) predicted that the interaction between the X-loop of NBD1 and CH4 may change with phosphorylation and initiation of the gating cycle of CFTR.

##### Disease-causing Mutations in the CHs of ICL1 and ICL4 Attenuate the Rate of CFTR Channel Activation

**FIGURE 6.**
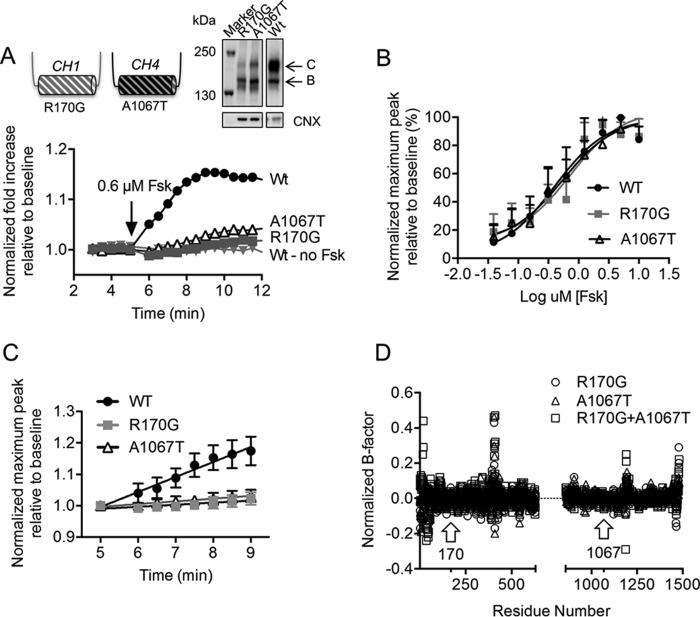
**PKA-dependent channel activation and flexibility of NBD1 are affected by disease-causing mutations at CH1 and CH4.** *A*, representative FLIPR traces of WT-CFTR (WT) and disease-causing ICL mutants, R170G on CH1 and A1067T on CH4, stimulated with 0.6 μm Fsk were normalized to unstimulated (*WT - no Fsk*) traces. *Inset*, immunoblot shows that the abundance of complex-glycosylated protein (band *C*) compared with core-glycosylated protein (band *B*) of the disease-causing loop mutants was significantly lower than WT-CFTR. *B*, the maximum peak at each forskolin concentration was normalized to the unstimulated trace and highest forskolin stimulation (10 μm) to generate the forskolin dose-response curves. The EC_50_ values were quite similar: 0.40 μm for WT, 0.73 μm for R170G, and 0.54 μm for A1067T. *Error bars* indicate ± 1 S.D. between replicate samples (*n* = 12 biological replicates and *n* = 4 technical replicates). *C*, from these calculated EC_50_ values, the closest forskolin concentration from our assay to these EC_50_ values was 0.6 μm. The initial slopes of activation within 4.5 min of forskolin stimulation with 0.6 μm forskolin were then compared. At 0.6 μm, the slopes of activation of the disease-causing ICL mutants were significantly lower than WT; the slope of activation for WT was 0.048 ± 0.002, whereas the slope of activation for R170G was 0.007 ± 0.001 and for A1067T, 0.009 ± 0.001. *Error bars* indicate ±1 S.D. between replicate samples (*n* = 12 biological replicates and *n* = 4 technical replicates). *D*, normalized temperature factor (B-factor) values, which represent protein flexibility, of the residues of R170G (*circles*) and A1067T (*triangles*) and both mutations, R170G+A1067T (*squares*), were determined by the protein structure prediction server, I-TASSER (30–33) and subtracted from WT. Interestingly, the disease-causing mutations (*arrows* showing residue numbers of the mutations) did not affect the B-factor of the CHs but significantly increased the B-factor at the N-terminal region of the regulatory insertion (residues 405–407) of NBD1.

Multiple disease-causing mutations have been identified in the CHs of ICL1 and ICL4, including R170G and A1067T, respectively (29). Both R170G and A1067T were predicted to reduce the helical propensity of ICL1 and ICL4 with secondary structure prediction software (data not shown) and were shown to impair the biosynthetic maturation of the CFTR protein (Fig. 6*A*, *inset*). These findings support the hypothesis that both CHs are important for CFTR assembly.

To determine if these helix-disrupting mutations also impair phosphorylation-regulated channel activity, we compared their forskolin sensitivity and kinetics of channel activation relative to the WT-CFTR protein. Given the lower steady-state levels of mature CFTR protein exhibited by R170G and A1067T (Fig. 6*A*, *inset*), it was not surprising to find that the peak of forskolin-mediated channel activity measured using the FLIPR assay was reduced for both mutant proteins relative to WT-CFTR (Fig. 6*A*). However, the dose response for forskolin-dependent activation was similar for the two mutants and WT-CFTR protein (Fig. 6*B*). This result suggests that these mutants do not exhibit a defect in forskolin-mediated phosphorylation *per se*. On the other hand, the rates of activation with forskolin (added at 0.6 μm, the forskolin concentration closest to the half-maximal effective concentration, EC_50_, of all the constructs) were significantly attenuated relative to the WT-CFTR protein for both CH mutants (Fig. 6*C*). We interpret these findings to suggest that both CHs are required for phosphorylation-dependent activation of the channel gate.

We used a structure prediction method called I-TASSER (30–33) to test our prediction that both of the mutations in CH1 and CH4 are disruptive to the transmission interface comprising NBD1. I-TASSER modeled the full-length CFTR sequence by generating and clustering structural simulations with the SPICKER program and then aligning the transmembrane region to similar PDB structures of ABC transporters that include P-glycoprotein, TM-0287, Atm1, PCAT1, and MsbA (30–33). We generated separate models of the full-length protein bearing R170G, A1067T, or both mutations (R170G+A1067T) for comparison. In Fig. 6*D*, we show the change in the normalized temperature factor (B-factor), an indication of protein flexibility, at all of the residues of the full-length mutant CFTR proteins relative to the WT protein (excluding the R region, as it is extremely flexible and can mask the effect of other structured regions of CFTR). The B-factor values were generated by an algorithm in I-TASSER known as ResQ which takes into account the changes in local structural assembly along with the sequence and structural profiles, and then these values were normalized based on the z-scores (34). Interestingly, the models for all of the three mutant proteins exhibited the largest change in B-factor in regions other than the CHs. Rather, the largest change for both mutants was detected at the N-terminal region of the RI (residues 405–407) of NBD1 (Fig. 6*D*). These models suggest that both of these disease-causing mutations in CH1 or CH4 act allosterically to induce similar defects in the conformational stability of NBD1. Together with channel activity studies, this modeling supports the role of the transmission interface in mediating phosphorylation-dependent activation.

## Discussion

The molecular mechanisms underlying the activation of CFTR channel function by PKA-dependent phosphorylation, ATP binding, and hydrolysis remain poorly understood mainly due to the paucity of structural information regarding the full-length protein. Hypothetical models for CFTR channel activation have been developed on the basis of patch clamp studies (35–37), cross-linking studies of cysteine-substituted CFTR in cellular membranes (8, 10, 38), and biophysical studies of isolated domains (4, 7). In this work, the first spectroscopic studies of purified full-length CFTR together with cell-based studies of disease-causing mutants support the importance of interdomain interactions between the MSDs and NBDs in phosphorylation-dependent channel activation. These findings provide a molecular framework that is important for advancing our understanding of regulated CFTR channel gating, the defects caused by CF-causing mutations, and potentially the mechanism of action of small molecule modulators of CFTR channel function.

Although the interaction between the MSDs and NBDs of other ABC proteins (*i.e.* solute transporters) is thought to form a structural conduit important for regulating transport function (39–41), previous chemical cross-linking studies failed to detect a change in the proximity of these domains in CFTR, a unique ABC channel protein, after phosphorylation, a necessary trigger for channel activity (1, 8). The present studies monitoring intrinsic tryptophan fluorescence in the purified full-length CFTR protein are the first to provide evidence supporting a model wherein phosphorylation modifies the affinity for coupling between the MSDs and NBDs of CFTR. Intrinsic tryptophan fluorescence studies showed that the propensity for urea-induced unfolding and fluorescence quenching by potassium iodide was reduced in the purified and highly phosphorylated CFTR protein relative to the basally phosphorylated protein. The water-accessible junction between the ICLs extending from the MSDs and the NBDs harbors multiple tryptophans and constitutes the region most likely to be modified by phosphorylation. The inability to detect changes in steady-state chemical cross-linking in our current work and previous studies (1, 8) supports the claim that the CHs are always in close physical proximity to the NBDs in both the basally and highly phosphorylated CFTR protein. Our findings suggest that the affinity of this interaction is increased with phosphorylation.

The importance of the transmission interface involving NBD1 (conferred by both CH1 and CH4) was substantiated in cell-based studies showing altered phosphorylation-dependent channel activation for two disease-causing mutations localized in these helices. Interestingly, rather than altering the sensitivity for the agonist of phosphorylation (forskolin), these mutations attenuated the kinetics of phosphorylation-dependent activation which implicates a defect in a secondary conformational change necessary of channel opening. Together, these data support a model wherein the interaction between the MSDs and NBDs of CFTR is modified by phosphorylation, and disruption of this transmission interface alters the kinetics of long range conformational changes vital for channel opening.

These studies are relevant to our understanding of the impact of the major mutant, deletion of phenylalanine at position 508 (F508del), on CFTR channel function. This mutation, located in NBD1 at the interface where CH4 binds, is known to disrupt CFTR assembly, biosynthetic processing, and phosphorylation-dependent channel gating (27, 42, 43). Relevant to the current work, it was previously reported that the rate of phosphorylation-dependent channel activation is attenuated in F508del-CFTR (44, 45), a similar phenotype that we have described for the disease-causing mutations in CH1 (R170G) and CH4 (A1067T). Given our finding that both interfaces (constituted by CH1 and CH4) contribute to normal channel activation by phosphorylation, we hypothesize that small molecule stabilizers of the ICL1-NBD1 interface may augment the efficacy of modulators that partially rescue the defect in the ICL4-NBD1 interface caused by F508del (*i.e.* Class I correctors). This concept was tested in part by Ehrhardt *et al.* (11) as they developed activators and inhibitors of CFTR channel activity by screening for enhancers or disruptors of the interaction of NBD1 with peptides derived from ICL1. In future studies we would include the CH region of the ICL1 peptides that was absent in the peptides tested by Ehrhardt *et al.* (11), as our study shows that this region will be particularly effective in identifying modulators of phosphorylation-dependent activation in WT-CFTR and potentially F508del-CFTR.

Finally, the biophysical methods described in this work will also be useful in future studies aimed at elucidating the mechanisms underlying the activity of small molecule modulators, especially potentiators that enhance CFTR channel activity. We have previously shown that the potentiator, ivacaftor (VX-770), acts directly on the phosphorylated CFTR channel protein to enhance its ATP-dependent and -independent channel openings (12). It will be interesting to determine if VX-770 binding enhances phosphorylation-dependent modification of the transmission interface by monitoring changes in intrinsic tryptophan fluorescence.

## Experimental Procedures

### 

#### 

##### Purification of Full-length WT-CFTR

Full-length WT-CFTR with a C-terminal polyhistidine (His_10_) tag was overexpressed in *Sf9* cells, and crude membranes were prepared as previously described (12). Crude membranes were solubilized by 2% fos-choline-14 (Anatrace, Maumee, OH). CFTR was purified via affinity to Ni-NTA column and exchanged to DDM (BioShop Canada Ltd., Burlington, Ontario, Canada) detergent micelles. The purified protein sample was run on a 4–12% SDS-acrylamide gel and developed by the silver stain method.

##### Synchrotron Radiation Circular Dichroism Spectroscopy

Full-length CFTR protein was purified in buffer (20 mm sodium phosphate, 25 mm sodium chloride, 1 mm DDM, pH 7.2). The protein sample was split into two conditions: non-PKA-phosphorylated and PKA-phosphorylated CFTR. In the PKA-phosphorylated condition, purified CFTR was phosphorylated with 200 nm PKA (New England Biolabs Ltd., Ipswich, MA) and 5 mm Mg-ATP (Sigma) in buffer (20 mm sodium phosphate, 25 mm sodium chloride, 1 mm DDM, pH 7.2) on the Ni-NTA column (Qiagen, Hilden, Germany) as previously described (12). PKA (38 kDa) was effectively washed of the PKA-phosphorylated sample with buffer (20 mm sodium phosphate, 25 mm sodium chloride, 1 mm DDM, pH 7.2 buffer) and a centrifugal filter with a cutoff of 100 kDa (Merck Millipore Ltd., Tullagreen, Ireland) as previously described (12). The non-PKA-phosphorylated sample was also washed the same way as the PKA-phosphorylated CFTR sample. SRCD spectra were obtained at the ISA synchrotron (Aarhus, Denmark) using a 0.1-mm path length sealed Suprasil (Hellma Analytics, Müllheim, Germany) quartz cuvette. For each spectrum a step size of 1 nm and an averaging time of 2 s were used. In each case, three replicate scans of the sample were subtracted from three scans of the baseline (20 mm sodium phosphate, 25 mm sodium chloride, 1 mm DDM, pH 7.2 buffer) and the net spectrum was calibrated to a spectrum of camphorsulfonic acid (CSA, Sigma) measured before data collection (46). Two sample loadings, each with three replicate scans, were conducted for each condition (non-PKA and PKA-phosphorylated). Delta epsilon (Δϵ) curves were scaled as previously described (47). All processing was carried out using the CDTool software (48). Differences were identified from *error bars* (indicating reproducibility levels) at all wavelengths, set at 1 S.D.

##### Intrinsic Tryptophan Fluorescence

Purified full-length WT-CFTR was concentrated to concentrations between 50 and 70 μg/ml as determined by the NanoDrop 2000 (1 Ab ≈ 1 mg/ml, ThermoFisher) in buffer (25 mm HEPES, 25 mm sodium chloride, 1 mm DDM, pH 7.2). Protein sample was split into two conditions: non-PKA-phosphorylated and PKA-phosphorylated as previously described. Intrinsic tryptophan fluorescence studies of the purified protein were conducted on the Photon Technology International QM80 spectrofluorimeter (HORIBA Scientific, Edison, NJ) with bandwidths of 2 nm for both excitation and emission and using a 10 × 2-mm quartz cuvette (Hellma Analytics). Fluorescence traces were corrected for dilution, inner filter effect, scattering and background (via subtraction of the buffer fluorescence trace).

##### Urea Denaturation Studies

Samples of 130 μl were then incubated at each urea concentration for 10 min at room temperature. Tryptophan residues were excited at 290 nm, and emission scans were run from 300 to 400 nm. The λ_max_) at each urea concentration was determined and plotted against the urea concentration with GraphPad Prism (version 6.0c). Sigmoidal curves of λ_max_ were fitted to the following equation using GraphPad Prism,

(Eq. 1) $$f\left(C\right)=\left(\exp\left(-m\left(C_{m}-C\right)/RT\right)/\left(1+\exp\left(-m\left(C_{m}-C\right)/RT\right)\right)\right)$$

where *m* is the *m* value or slope of the urea denaturation curve, *C* is the individual urea concentration, and *C_m_* is the midpoint urea concentration (49). 3 μm NATA (Sigma) was used as a control in which all the tryptophan residues were exposed to the solvent environment.

##### Quenching Studies

Purified CFTR samples were titrated with 2-μl titrations of 2.5 m stock concentrations of acrylamide, potassium iodide, or cesium chloride quenchers dissolved in buffer (25 mm HEPES, 25 mm sodium chloride, 1 mm DDM, pH 7.2). Fluorescence intensity at each quencher concentration was taken over a 20-s interval by excitation at 290 nm and emission at 322 nm. Fluorescence intensities were averaged over the 20-s interval and corrected for background. Values of initial fluorescence intensity of purified CFTR without quenchers (*F*_0_) over fluorescence intensity at each quencher concentration (*F*) were plotted at each quencher concentration ([*Q*]). This plot known as a Stern-Volmer plot generates a linear regression using GraphPad Prism with the following equation.

(Eq. 2) $${\text{F}}_{0}/\text{F}=1+K_{\text{SV}}\left[Q\right]$$

The Stern-Volmer constant (*K*_sv_) was determined as the slope of each linear regression.

##### Generation and Expression of Mutants

The full-length WT-CFTR construct with all native cysteine residues mutated to other residues (cys-less WT-CFTR), the cys-less WT-CFTR with V510C at NBD1, and A1067C at CH4 on separate plasmids and on the same plasmid (V510C/A1067C) were kindly provided by Dr. David Clarke (Toronto, Ontario, Canada) (26). To probe the ICL1-NBD1 interface, primers of R170C (forward, 5′-CTT TAA AGC TGT CAA GCT GTG TTC TAG ATA AAA TAA G-3′, and reverse, 5′-CTT ATT TTA TCT AGA ACA CAG CTT GAC AGC TTT AAA G-3′), W401C (forward, 5′-GTA ACA GCC TTC TGT GAG GAG GGA TTT GG-3′, and reverse, 5′-CCA AAT CCC TCC TCA CAG AAG GCT GTT AC-3′) and E476C (forward, 5′-GAT TAT GGG AGA ACT GTG TCC TTC AGA GGG TAA AAT TAA G-3′, and reverse, 5′-CTT AAT TTT ACC CTC TGA AGG ACA CAG TTC TCC CAT AAT C-3′) were designed to generate single and double cysteine mutants on the cys-less WT-CFTR cDNA template that was kindly provided by Dr. David Clarke (26). Primers of disease-causing loop mutants, R170G (forward, 5′-GAC TTT AAA GCT GTC AAG CGG TGT TCT AGA TAA AAT AAG-3′, and reverse, 5′-CTT ATT TTA TCT AGA ACA CCG CTT GAC AGC TTT AAA GTC-3′) and A1067T (forward, 5′-GGA CAC TTC GTA CCT TCG GAC GG-3′, and reverse, 5′-CCG TCC GAA GGT ACG AAG TGT CC-3′), were generated on full-length human WT-CFTR cDNA (pcDNA3.1). Mutations were generated using the KAPA HiFi HotStart PCR Kit (KAPA Biosystems, Woburn, MA), and plasmid DNA was generated with the QIAprep Spin Miniprep kit (Qiagen). Mutations were confirmed with DNA sequencing (TCAG, Toronto, Ontario, Canada). Plasmid DNA of mutants were transfected using Polyfect Transfection Reagent (Qiagen) in HEK-293 GripTite cells kindly provided by Dr. Daniela Rotin (Toronto, Ontario, Canada). Transfected cells were then temperature-rescued at 27 °C for 24 h.

##### FLIPR Membrane Potential Assay

The following steps of the FLIPR assay were conducted at 27 °C. Cells were loaded with 0.5 mg/ml FLIPR membrane potential dye (Molecular Devices, Sunnyvale, CA) in sodium gluconate buffer (140 mm sodium gluconate, 0.5 mm potassium gluconate, 2 mm calcium gluconate, 2 mm magnesium gluconate, 10 mm HEPES, 12 mm sodium bicarbonate, pH 7.4) for 45 min. Fluorescence was recorded at an excitation of 530 nm and emission of 560 nm on the fluorescence plate reader (SpectraMax i3X, Molecular Devices). Baseline fluorescence was read for 5 min, and stimulation of CFTR activity by 10 μm forskolin (Sigma) was read for up to 15 min. The maximum peak of activation and the maximum rate during the first 5 min of forskolin stimulation, which was determined by linear regression with GraphPad Prism, were analyzed.

##### Cysteine Cross-linking

Cells transfected with cys-less WT-CFTR constructs were pretreated with the cAMP agonists, 10 μm forskolin, and 100 μm 3-isobutyl-1-methylxanthine (Sigma) or vehicle (DMSO) for 30 min at 27 °C. 50 μm BMOE (Life Technologies) in Dulbecco's modified Eagle's medium media (Wisent, St-Bruno, Quebec, Canada) was added to HEK-293 GripTite cells transfected with the cys-less WT constructs for 1 h on ice as previously described (10). Transfected HEK-293 GripTite cells were lysed with radioimmunoprecipitation assay buffer (50 mm Tris base, 150 mm sodium chloride, 1 mm EDTA, 1% (v/v) Triton X-100, 0.1% (v/v) SDS, 1× protease inhibitor mixture (AMRESCO, Cleveland, OH), pH 7.4). Protein samples were run on 6% Tris-glycine sodium dodecyl sulfate gels (Life Technologies) and transferred onto nitrocellulose paper. Immunoblots were probed for total CFTR protein with human CFTR NBD2 specific (amino acid 1204–1211) IgG2b mAb596 antibody (1:1000, University of North Carolina at Chapel Hill, Chapel Hill, NC, code: A4) overnight at 4 °C and horseradish peroxidase-conjugated goat anti-mouse IgG secondary antibody (1:2000, Pierce) for 1 h at room temperature. Immunoblots were probed for phosphorylated CFTR protein with phosphorylation-sensitive (anti-Ser-813, quenched by PKA phosphorylation) IgG1 mAb217 antibody (1:1000, University of North Carolina at Chapel Hill, code: A3) overnight at 4 °C and horseradish peroxidase-conjugated goat anti-mouse IgG secondary antibody (1:2000, Pierce) for 1 h at room temperature. The specificities of these primary antibodies on CFTR epitopes were previously described (8, 50, 51). Protein loading was normalized with the calnexin-specific rabbit pAb (1:10,000, Sigma, catalog number C4731) primary antibody overnight at 4 °C and horseradish peroxidase-conjugated goat anti-rabbit IgG secondary antibody (1:10,000, Pierce) for 1 h at room temperature. Blots were exposed with Amersham Biosciences enhanced chemiluminescent reagent (GE Healthcare) on the Li-Cor Odyssey Fc (LI-COR Biosciences, Lincoln, NE) in a linear range of exposure. Normalized densitometry of immunoblot bands to total protein signal were analyzed with ImageJ 1.48v (imagej.nih.gov).

##### Expression and Function of Disease-causing Loop Mutants

Immunoblotting of disease-causing loop mutants was performed as previously mentioned with the exception that the immunoblots were probed with human CFTR NBD2 IgG2b mAb596 antibody (1:2000, University of North Carolina, code: A4) overnight at 4 °C and horseradish peroxidase-conjugated goat anti-mouse IgG secondary antibody (1:4000, Pierce) for 1 h at room temperature. The EC_50_ values of phosphorylation-dependent channel activation of WT, R170G, and A1067T were determined by activating the channel with various forskolin concentrations (i.e. 0, 0.08, 0.16, 0.31, 0.63, 1.25, 2.5, 5, and 10 μm) and normalizing the maximum peak of activation to no forskolin control in the FLIPR membrane potential assay. Curves were fit with the dose-response stimulation log (agonist) *versus* response (three parameters) fit in GraphPad Prism. Slopes of activation within 4.5 min after 0.6 μm forskolin (the forskolin concentration closest to all the EC_50_ values of WT and the disease-causing ICL mutants) were determined by linear regression on GraphPad Prism.

##### Bioinformatics

The locations of CH residues, Arg-170 and Ala-1067, were identified from the Dalton-Kalid homology model (17) using the molecular graphics package, PyMOL (53). The secondary structure predictions of WT-CFTR and the mutants (R170G, A1067T, and R170G+A1067T) were conducted using the I-TASSER server (30–33). The ResQ algorithm of I-TASSER generated the B-factor values by taking into account the changes in local structural assembly along with the sequence and structural profiles (30–33). These values were then normalized based on the z-scores (30–33).

## Author Contributions

S. C. was the primary contributor to the design, performance, and analysis of experiments shown in Figs. 1 and 3–6. D. W. assisted in the design and performance of mutagenesis studies and channel activity measurements. A. J. M. contributed to design, performance, and analysis of SRCD studies. P. D. Y. E. contributed to the development of CFTR purification methods and interpretation of intrinsic fluorescence studies. S. V. M. contributed to the design and performance of mutagenesis studies. B. A. W. provided advice regarding the performance of SRCD and oversaw analysis of results. C. E. B. oversaw the development of the research plan and contributed to the interpretation of data and writing of the manuscript. All authors reviewed the results and approved the final version of the manuscript.

## Footnotes

2 The abbreviations used are:

CFTR: cystic fibrosis transmembrane conductance regulator
NBD: nucleotide binding domain
CH: coupling helix
ICL: intracellular loop
R domain: regulatory domain
MSD: membrane spanning domain
RI: regulatory insertion
Sf9: *Spodoptera frugiperda*
Ni-NTA: nickel nitrilotriacetic acid
DDM: *n*-dodecyl β-d-maltoside
SRCD: synchrotron radiation circular dichroism
λ_max_: emission wavelength maximum
NATA: *N*-acetyl-l-tryptophanamide
*C_m_*: midpoint urea concentration
*m* value: rate of free energy change with urea concentration
*K*_sv_: Stern-Volmer constant
cys-less: cysteine-less
BMOE: bismaleimidoethane
FLIPR: fluorescence imaging plate reader
Fsk: forskolin
I-TASSER: Iterative Threading ASSEmbly Refinement
B-factor: temperature factor
F508del: deletion of phenylalanine at position 508.

## Addendum

A full-length structural model of the zebrafish CFTR was recently defined by cryo-microscopy (52). The model represents the closed state of the channel as it is dephosphorylated and ATP free. The R domain in this model lacked stable secondary structure, but interestingly, it did confer an amorphous density that extended from the inner vestibule of the channel pore all the way down the long axis conferred by ICL1, ICL4, and the ATP binding surface of NBD1. Hence, the zebrafish CFTR structure is compatible with the idea that phosphorylation of the R domain is poised to indirectly modify the transmission interface between the CHs of ICL1, ICL4, and NBD1.
